# Reproductive and sexual Health of surrogate mothers, developing a care program: a protocol for mixed methods study

**DOI:** 10.1186/s12978-019-0687-8

**Published:** 2019-02-26

**Authors:** Marjan Goli, Ziba Farajzadegan, Zeinab Heidari, Shahnaz Kohan

**Affiliations:** 1 Reproductive and sexual Health, Faculty of Nursing and Midwifery, Isfahan University of Medical Sciences, Isfahan, Iran; 2 Nursing and Midwifery Sciences Development Research Center, Najafabad Branch, Islamic Azad University, Najafabad, Iran; 3 Department of Community and preventive medicine, Medicine Faculty, Medical Sciences University Of Isfahan, Isfahan, Iran; 4 Reproductive and sexual Health, Nursing and Midwifery Care Research Center, Faculty of Nursing and Midwifery, Isfahan University of Medical Sciences, Isfahan, Iran

**Keywords:** Gestational surrogacy, Reproductive and sexual health, Surrogate mother, RAND method, Mixed method, Protocol for study

## Abstract

**Background:**

Gestational surrogacy is one of the options for women whom pregnancy is contraindicated. Despite of increasing demand for gestational surrogacy, its various aspects are controversial. The unique nature of surrogacy causes surrogate mothers to face a variety of problems such as, ethical confusion, psychological disturbance and reproductive health matters. Therefore, it is necessary to develop a comprehensive care program for reproductive and sexual health providing specific care at prenatal and pregnancy as well as delivery and postpartum period in socio- cultural context of Iran.

**Methods:**

This research is an exploratory study with the qualitative-quantitative sequencing design (mixed) that is consisted of three sequential phases. In the first phase, following a qualitative approach, the researcher will explain needs of reproductive and sexual health in surrogate mothers. In the second phase, a primary reproductive and sexual health care program is designed for surrogate mothers in which, in addition to using the qualitative study results, related papers and texts will be also used. In the third phase of the study, reproductive and sexual health care program will be evaluated by RAND method (RAM).

**Discussion:**

The results of this mixed method study are expected to lead to the development of a reproductive and sexual health care program meeting the needs of surrogate mothers and are in accordance with the cultural conditions of the research community, ultimately leading to improvement in reproductive and sexual health of surrogate mothers.

## Plain English summary

Fertility and family are considered the most basic foundations of life, and infertility is one of the greatest problems of the humans’ lives. Surrogacy is one of the assisted reproductive techniques which involve using the third party. Despite the increased use of surrogacy, this treatment method is associated with complexities. Reproductive and sexual health care of surrogate mothers has been not discussed clearly in the textbook of obstetrics and there are few clinical practice guidelines on this topic. Considering lack of a specific reproductive and sexual health care program for surrogate mothers in Iran and also the challenges that these women would face, the findings of this study are suitable sources of information for the required interventions to promote the reproductive and sexual health of them. This study is an exploratory study with the qualitative-quantitative sequencing design (mixed) that is consisted of three consecutive phases. Following a qualitative approach, the researcher will explore the needs related to reproductive and sexual health of surrogate mothers. At the second phase, a primary reproductive and sexual health care program will be designed in which, in addition to using the qualitative study results, related papers and texts will be also used. In the third phase, validating of the reproductive and sexual health care program will be investigated by RAND method. Therefore, conducting a mixed methods study is expected to design a reproductive health program for surrogate mothers in order to improve their reproductive and sexual health status and wellbeing.

## Background

In most cultures, fertility is considered as a significant value and the hope for having a child is considered as one of the basic motivators in the humans [1]. So family and fertility are considered the most fundamental pillars of life, and infertility is one of the greatest problems of the humans’ lives [2]. According to a report by the World Health Organization (WHO), about 80 million people (about 10 to 15%) are entangled with infertility problem, worldwide [3]. Based on the global statistics indicating the rate of 10 to 15%, the number of infertile couples in Iran is about 3 million people (1.5 million couples) and about 100,000 infertile couples would be added to this number annually [4].

Since the ancient times, humans have made various efforts to overcome infertility. These methods have been found in response to the needs of infertile couples [2]. Wherever the conditions or diseases lead to inability to gestate a pregnancy, third party reproduction could be considered as an option. This option is called as gestational surrogacy [5]. Meanwhile, gestational surrogacy could be one of the methods for treating infertility. This method is in the form of an agreement, in which the woman agrees to carry a pregnancy and relinquish the child to the intended couple at the birth time [6].

The most indicators necessitating for gestational surrogacy (GS) are congenital absent or abnormal uterus, hysterectomy, Mullerian anomaly, unexplained recurrent miscarriages, repeated failures in infertility treatments, failure in embryo implantation, maternal medical conditions when pregnancy would increase the risks to her health, Use of teratogenic drugs by the mother to treat the disease and finally a poor obstetric history [7].

The unique nature of gestational surrogacy causes surrogate mothers to face a variety of problems regarding pregnancy and delivery compared to other women [8]. Reproductive and sexual health care of a surrogate mother has been not discussed clearly in the standard textbook of obstetrics and there are limited clinical practice guidelines on this topic. As the surrogacy is becoming more common, it is necessary for providers of the reproductive health care to be conscious of the related ethical, legal and birth issues. This phenomenon has led to public discussion about the possible consequences regarding surrogacy such as economic exploitation, ethical confusion and psychological damages to the surrogate mother [9].

Surrogacy has more controversies in aspects such as medical, legal and psychological screening, counseling, antenatal care and delivery process, legal, breastfeeding and psychological issues after birth. All of these matters require following a comprehensive approach between the entire participants including obstetricians, gynecologist, midwives, psychologists, lawyers, surrogate mothers and intended couples, infertility clinics, facilitating agencies and the hospitals [7].

The use of surrogacy has been increased in the United States and it consists about 2.5% of all the assisted reproductive treatments (ARTs) in 2013 [10] . From 2007 to 2012, the number of children who were born through surrogacy was increased by 91.5%. Surrogacy has become a more common reproductive option [11] for the increasing infertile population of Canada.

In Iran, use of surrogacy was conducted first in 2001 by some of the infertility clinics [3] .While this issue is not involved in any specific law [6], its use is increasing [12]. As an option, this method has attracted the attention of many Iranian infertile couples, but it has been associated with many social and ethical issues which requires using a comprehensive approach in terms of its negative and positive aspects [13].

Although, there are no specific laws about surrogacy in Iran, Iranian couples are becoming more willing to use this method, and since this issue is considered as a taboo, gestational surrogacy is performed secretly, therefore providing sexual and reproductive health cares for these women would become ambiguous. In fact, health-determinant social factors could influence greatly on receiving reproductive and sexual health services in these women [14] .

Although surrogacy has been performed since the 1980s, it is still considered as a new method which requires evaluation, policy making, programming and comprehensive and deep introduction from different aspects and each of the fields of science should take part in illuminating its different aspects. In fact, when it comes to surrogacy, besides considering the scientific, medical and even legal aspects of the matter, it is important to consider the feelings, experiences, and concerns of women who take part in this method [15].

Considering lack of a specific reproductive and sexual health care program for these women in Iran and also the challenges that these women would face, the present study is designed to evaluate the needs of surrogate mothers and the opinions which are available for the experts and policy maker on providing care for these mothers, to develop a reproductive and sexual health care program.

### Objectives

Objectives of each phase are as the following:

#### Objectives of the first phase: Qualitative study

Exploring reproductive and sexual health needs of a surrogate mother.

#### Objectives of the second phase: Program designing

Designing a preliminary care program, based on the needs extracted from the results of the qualitative study and reviewing the literature.

#### Objectives of the third phase: Quantitative study

Evaluating the developed reproductive health care program for surrogate mothers by RAND appropriateness method (RAM).

## Methods

This study is an exploratory study with the qualitative-quantitative sequencing design (mixed) that is consisted of three sequential phases. Following a qualitative approach, the researcher will explore the needs of surrogate mothers from the viewpoint of surrogate mothers, health care providers and policy makers. Data will be collected through deep, semi-structured interviews and taking field notes. Participants who are eligible for participating in the study will be selected in a purposive manner and with maximum variety. While collecting the data, the interviews will be analyzed using a conventional qualitative content analysis method simultaneously. Sampling will be continued until data saturation occurs. At the second phase of the study, a reproductive and sexual health care program will be designed for the surrogate mothers, using the results of qualitative study and reviewing the literature. Then, this proposed care program will be validated by RAND appropriateness method (RAM).

### First phase: Qualitative study

The first phase of the present study was designed to answer the following question “What are the reproductive and sexual health needs of surrogate mothers?” This study was carried out using qualitative content analysis method.

#### Sampling method

Participants will be selected by a purposeful sampling method and with maximum variety regarding age, education, social status, and job.

#### Characteristic of participants

Women who had experienced surrogate pregnancy and also care providers who had the experience of providing health and medical services for surrogate mothers, including gynecologist, psychologists and psychiatrists, obstetricians, midwives, maternal health policy makers, with at least 1 year of working experience in this field, will be considered as the study population who would participate in the present study voluntarily and with informed consents.

#### Research setting

Interviews will be conducted at the time and place designated by them (hospital, health centers, work places, university, home, etc.) and in agreement with the participants.

#### Data collection process

After taking the necessary permissions and sending introduction letters, the researchers would refer to the study settings, and after providing complete explanations about research goals, purposeful sampling will be started. Data collection methods include individual, in-depth and semi-structured interviews and field noting. Then participants will assigned an appointment in a private and comfortable environment, the time and place of the interview would be decided in agreement with the participants. The researchers will ensure that the participants are free to discontinue their cooperation with the study whenever they want. The duration of the interviews would be determined based on the participants’ information and willingness. After explaining objectives and methodology of the study, the researcher will receive written consents regarding participation in the research, further interviews, and recording the interviews. If a person does not agree with recording, taking notes will be done.

In the semi-structured individual interviewing method, each interview will be started by asking an open-ended question about the intended interviewee’s reproductive health needs and the effects of surrogacy on her reproductive health. Next questions will be asked based on her answers to the first question and also based on the interview guide. Whenever needed, we will also use probing questions such as “What do you mean by this?” “Can you provide more detailed explanations?” “What did you feel about this topic?” Moreover, at the end of each interview, the interviewer will be allowed to speak about any missed points.. During interviews, nonverbal messages of participants (such as tone, silence, emphasis, cry, and sigh) will also be documented.

After the end of each interview, the interview will be transcribed at the earliest opportunity and data analysis will be carried out simultaneously with data collection. Data collection will be continued until the data saturation is reached, that is, when no new data is extracted.

#### Data analysis

Data will be analyzed using conventional content analysis [14]. After recording the interviews, the researcher will change the recordings into texts regularly. Then, the written texts will be reviewed repeatedly to achieve a comprehensive understanding of the interviews. Analysis of the data achieved from the qualitative stage in the present study would be started simultaneous to the end of the first interview using conventional content analysis. So, the interviews would be considered as the content for analysis (first step: determining the content of analysis), the essence of the phrases and key words would be named (second step: primary coding), and then the primary codes would be merged and filtered and would be categorized based on their achieved meanings (primary categorization of the data). Following, categories that suggest similar concepts would be merged and subcategories would be formed (formation of the subcategories). Eventually, using inductive method and constant comparison, the main categories would be formed.

#### Rigor and trustworthiness of qualitative data

In order to assure the rigor of the data in the present study, four suggested criteria would be used [16] introduced by Streubert and Carpenter (2011) (including credibility, dependability, transferability or fittingness and conformability). Using the methods such as reviewing the interviews by the participants, peer review by the researcher’s colleagues, considering maximum variation in selecting the participants, spending sufficient time for data collection, and the integrating multiple data collection methods, such as interviewing and taking notes in the field, the accuracy and reliability of the qualitative data would be improved.

Credibility of the data will be approved through constant involvement with the data of the study, data coding simultaneous with data collection and participants’ approval, and using the corrective suggestions by the colleagues. In order to approve the conformability of the data, the opinions of researchers would be sought outside the settings of the present study and the stages of the study would be recorded accurately and the continuity of the work would be controlled regularly. By merging the collected data from in-person interviews and in-field notes, dependability of the data would be approved and to approve the transferability of the data, reevaluation method will be used as well as the approval by individuals outside the settings of the present study who are similar to the participants.

### Second phase: Developing the care program

In the second phase of the study, by using the data obtained from the qualitative phase, following the reproductive health needs of surrogate mothers which were determined, and after the extracted needs were prioritized and the literature was reviewed, the draft would be developed for the reproductive and sexual health care for surrogate mothers.

Literature review in the present study would be conducted using narrative review method which includes searching through library resources (reviewing references and theses), and also searching electronic references to find the existing knowledge in the field of cares and the care needs of surrogate mothers before, during and after delivery. At this phase, all of the studies about surrogate mothers, outcome of surrogacy and clinical guidelines that have been published during 2000 and 2018 in English and Persian would be reviewed. In the next phase, the search is performed using keywords and different combinations including “surrogate mother” and “surrogacy” and “carrier mother” and “gestational carriers” and “gestational surrogacy” and “gestational surrogate”. Several databases are used to search and identify related articles, including PubMed, Science Direct, and Web of Science, Cochrane Library, Scopus, Pro Quest, Ovid, Magiran, SID, MEDLINE ،Embase، CINAHL and Google scholar.

### Third phase: Evaluating the developed reproductive health care program

In the third phase of the study, the developed reproductive health care program for surrogate mothers would be evaluated by RAND appropriateness method (RAM). This method would provide the opportunity for extension of appropriate criteria based on the existing evidence and would be completed using the opinions of experts. Studies that have evaluated RAND method (RAM) have concluded that it is a reliable and valid method for evaluating the appropriateness of the cares [17].

In the present study, to achieve consensus between the experts, (RAM) will be used in modified two-round Delphi method. In the first round, each of the care indications would be scored individually by the relevant experts and in the second round, rating would be performed in a face-to-face group discussion [18]. So, based on the qualitative study and by reviewing the literature, a list of cares would be developed in a table. Then the developed table would be sent to all of the members of Delphi including infertility specialists, psychiatrists, reproductive health specialists, psychologists, and maternal policy makers. At this round, the researcher would visit each of the Delphi members and explain the method of scoring for them. Then, they would be asked to rate each of the scenario from 1 to 9 based on their clinical judgment, by reviewing up-to-date articles and considering the ratio of benefits of the care to its risks. The rate of 1 means that it is expected that the risks of the care would be more than its advantages and the rate 9 means that it is expected that the benefits of the intended cares would be more than its risks. A rate of 5 means that the risks and benefits are not determined or their effects are equal. Eventually, each of the mentioned cares in the list would be labeled as “appropriate”: (mean score of 7 to 9), “undetermined”: (mean score of 4 to 6) or “inappropriate”: (mean score of 1 to 3) based on the ranking and scoring of the experts. Also, the experts are allowed to add scenarios to this list based on their own experiences and knowledge [19].

In this regard, in the second round of gathering, after making the coordination and sending invitations to the experts, a meeting would be held and the members of Delphi would be gathered under the leadership of researcher in the meeting. In this round, the experts would discuss regarding the priority for implementing the care programs while focusing on the conflicting cases and eventually each indication would be rated individually.

After gathering and evaluation of the indications by the members based on their consensus rate, they would be divided into three categories: appropriate, undetermined and inappropriate. [20].

In the end, considering the conclusions of the experts’ opinions and by performing necessary modifications, the final format of the reproductive and sexual healthcare program for surrogate mothers would be developed based on the needs, beliefs and culture of the studied population.

## Discussion

Surrogacy is one of the methods for building a family, in which a third party takes part for carrying the pregnancy; so a woman would become pregnant for a commission couple [21]. Surrogacy would be considered as the last resort for having a child in some of the infertile couples [22] and it is an alternative method for women who are not able to carry a pregnancy by any other methods [23] .

The use of surrogacy may cause new challenges in terms of the ethical, emotional, social and legal fields and if these issues are not addressed, this method could not be used with satisfaction [10]. Despite concerns about the long-term effects of surrogacy on surrogate mothers, studies are limited in this regard [24] .

Discrepancies regarding the laws of surrogacy indicate that this method is controversial. In many industrial countries such as France, Germany, Italy and Spain and some states of United States, surrogacy is banned. In some countries, such as England, Netherlands, Greece, Australia, and South Africa, only non-commercial and altruistic surrogacy is allowed. In Sweden, surrogacy is partly unregulated but the situation is under evaluation and might be changed [22].

To develop any guideline, the needs, values, beliefs and ethical, spiritual and cultural differences of the society should be considered. Needs assessment is the first and most fundamental step in programing the health system and medical education [25].

Therefore, needs assessment is an inoperable part of planning; during this process the needs would be determined, based on priorities, and measures would be conducted to fulfill them, and the data achieved from needs assessment would provide the necessary information for planning [26].

Considering the increased use of surrogacy and lack of a comprehensive reproductive and sexual health care program for these women, this study is trying to find the reproductive and sexual needs and develop a comprehensive care program that could explain health and the effective social factors on health. The results of this mixed methods study are expected to lead to the development of a reproductive and sexual health care program with Inter professional approach that meets the needs of surrogate mothers and is in accordance with the cultural conditions of the research community for improving the reproductive and sexual health status of surrogate mothers.

## Abbreviations

ARTs: assisted reproductive treatments
GS: Gestational surrogacy
RAM: RAND appropriateness method
